# Voltage oscillations and response dynamics in a model of sensory hair cells

**DOI:** 10.1186/1471-2202-13-S1-P186

**Published:** 2012-07-16

**Authors:** Alexander B Neiman, Kai Dierkes, Benjamin Lindner, Andrey L Shilnikov

**Affiliations:** 1 Department of Physics and Astronomy, Ohio University, Athens, Ohio 45701, USA; 2 Max Planck Institute for the Physics of Complex Systems, Dresden 01187, Germany; 3 Bernstein Center for Computational Neuroscience, Humboldt University Berlin, Berlin 10115, Germany; 4 Neuroscience Institute, Georgia State University, Atlanta, Georgia 30303, USA; 5 Department of Mathematics and Statistics, Georgia State University, Atlanta, Georgia 30303, USA

## 

Sensory hair cells in auditory and vestibular organs rely on active mechanisms to achieve high sensitivity and frequency selectivity with respect to weak stimuli. Self-sustained oscillations in hair cells occur on two very different levels. First, the mechano-sensory hair bundle itself can undergo spontaneous mechanical oscillations. Second, self-sustained electric voltage oscillations across the membrane of the hair cell have been documented in the inner ear of lower vertebrates. The functional significance of these self-sustained voltage oscillations is currently unknown.

We used a Hodgkin-Huxley type model of the baso-lateral ionic currents of bullfrog sacculus to study genesis of spontaneous voltage oscillation patterns and how the spontaneous oscillations shape the response of the hair cell to external mechanical stimuli. To examine the influence of inevitable fluctuations on the dynamical regimes, we included a stochastic transduction current originating in the Brownian motion of the hair bundle and channel noise arising due to the finite number of mechano-electrical transduction channels [1].

We determined the bifurcation structure of the model in terms of two important ionic conductances, associated with the inwardly rectifier (K1) and Ca2^+^-activated (BK) potassium currents. We found that for large values of BK conductance the system is either at equilibrium or exhibit tonic oscillations. For small values of BK and large values of K1 conductances the dynamics of the model shows diverse patterns of activity including quasi-periodic oscillations, large-amplitude periodic spikes, and bursts of spikes. In particular we found a peculiar transition to bursting through quasiperiodic oscillations with two independent frequencies corresponding to a 2D torus in the phase space of the system. Within small patches of parameter space at the transition from spiking to bursting and at the spike adding transition, voltage dynamics are chaotic. Furthermore, we showed that thermal fluctuations of mechano-electrical transduction current can lead to chaos in a wide area of parameter space.

We found a high sensitivity and frequency selectivity for the regime of regular spontaneous oscillations in response to sinusoidal stimuli with frequencies *f* > 5 Hz. Hence, an oscillatory voltage compartment might constitute a biophysical implementation of a high-gain amplifier. Cells poised in the chaotic regime of low BK and high K1 conductances showed poor tuning, but provided a high sensitivity to low-frequency variations of external stimuli. In summary, this study shows that the electrical oscillator found in saccular hair cells contributes significantly to nonlinear amplification of external mechanical stimuli. This further supports the idea of nonlinear oscillators playing a crucial role in the operation of the inner ear.
